# Early COVID‐19 Vaccine Initiation and Completion Among Cancer Survivors and Barriers to Vaccine Completion: Implications for Future COVID‐19 Vaccination Uptake Rates

**DOI:** 10.1002/cam4.70428

**Published:** 2024-11-25

**Authors:** Nikta Saeedi, Angel Arizpe, Katelyn J. Queen, Jennifer Tsui, Sue Kim, Brian Z. Huang, Albert J. Farias

**Affiliations:** ^1^ Department of Population and Public Health Sciences Keck School of Medicine of the University of Southern California Los Angeles California USA; ^2^ Gehr Family Center for Health Systems Science University of Southern California Los Angeles California USA; ^3^ USC Norris Comprehensive Cancer Center Los Angeles California USA

**Keywords:** All of Us, cancer survivorship, COVID‐19 vaccine

## Abstract

**Purpose:**

Cancer survivors are considered by public health officials as a high‐risk group in the United States for severe complications from COVID‐19. We aimed to characterize patterns of early uptake of the COVID‐19 vaccine among cancer survivors and to determine modifiable barriers to vaccine completion that can be addressed to ensure future booster adherence.

**Methods:**

Cross‐sectional data of vaccine uptake by summer 2021 was extracted from adult cancer survivors enrolled in the “All of Us” research program. Vaccine completion was determined based on receiving at least two doses. We assessed sociodemographic factors, socioeconomic barriers (education, income, health insurance status, housing, and employment status), and COVID‐19 vaccine uptake by Summer 2021, employing multivariable ordinal logistic regression for those who were unvaccinated, had initiated vaccine uptake, and had completed COVID‐19 vaccination.

**Results:**

Of the 514 cancer survivors in the sample, 73.7% were fully vaccinated by summer 2021. Those who received no vaccine doses showed higher proportions of SES barriers, medical distrust, and perceived lack of need barriers. Race (non‐Hispanic White vs. other) was not statistically significantly associated with vaccine uptake (OR (95% CI) = 0.94 (0.51, 1.70)), while for every additional SES barrier, there was a 40% decrease (OR (95% CI) = 0.60 (0.48, 0.75)) in the odds of receiving more COVID‐19 vaccine doses. Higher medical distrust and perceived lack of need were associated with 56% (OR = 0.44, 95% CI: 0.32–0.59) and 39% (OR = 0.61, 95% CI: 0.43–0.87) lower odds, respectively.

**Conclusion:**

Racial/ethnic disparities in vaccine uptake may be explained by SES barriers. Addressing SES disparities and fostering medical trust may enhance future COVID‐19 vaccination uptake rates for this high‐risk group.

## 1 Introduction

There are over 18.1 million cancer survivors living in the United States at heightened risk of mortality and of experiencing severe complications from Coronavirus disease 2019 (COVID‐19) [1]. Recognizing the vulnerability of cancer survivors, both the Centers for Disease Control and Prevention (CDC) and the National Comprehensive Cancer Network (NCCN) have identified cancer survivors as a high‐risk group for severe illness from COVID‐19 [3, 4]. In late 2020, the Pfizer‐BioNTech and Moderna/US NIAID vaccines were granted Emergency Use Authorization (EUA) and cancer survivors, especially those over 65 years of age, were among the highest priority group in order to lower their risk of contracting the virus and create safer environments for cancer patients to attend hospital appointments and receive necessary treatments [5].

As of May 2023, an estimated 69.5% of the US population completed a primary series of the COVID‐19 vaccine, and 17.0% received an updated booster dose [6]. With COVID‐19 becoming endemic, regular vaccination is crucial for high‐risk groups to protect against variants. However, more data are needed to determine vaccination rates among cancer survivors and identify subgroups for targeted interventions to increase routine vaccination.

During the first 6 months of COVID‐19 vaccine rollout beginning in December 2020, significant vaccine hesitancy was observed among cancer survivors, with studies indicating that 20%–37% reported reluctance or hesitancy toward receiving the COVID‐19 vaccine [7, 8, 9].

Vaccine hesitancy is shaped by structural inequalities and systemic discrimination that disproportionately affect individuals based on sociodemographic factors such as race/ethnicity, age, biological sex, education, and employment. These inequalities influence healthcare accessibility, risk perceptions, trust in health authorities, and beliefs in vaccine safety and efficacy [10], further contributing to disparities in vaccination uptake. Such patterns are observed in influenza vaccination rates, where persistent gaps exist with Black, Hispanic, and AIAN adults having significantly lower vaccination rates than White adults [11]. Research has also shown that social determinants of health also impact flu vaccine uptake where vaccine uptake is lower among African Americans who have lower knowledge about the flu vaccine, less trust in the vaccine, and perceive greater barriers to getting vaccinated [12]. Cancer survivors also harbor unique concerns regarding vaccination, including apprehensions about potential interactions with ongoing cancer treatments, fears of diminished treatment efficacy, and heightened side effects [13].

Vaccine hesitancy among cancer patients is further complicated by existing disparities in COVID‐19 burden and risk. Structural and social inequities within our healthcare system have led to a disproportionate burden of COVID‐19 on racial/ethnic minorities, individuals with lower socioeconomic status, and those facing barriers to healthcare access [14, 15, 16]. These disparities are more pronounced among underserved populations living with cancer, highlighting the need to study the factors influencing both primary and booster COVID‐19 vaccine uptake in this high‐risk group.

While prior research on risk factors associated with COVID‐19 vaccine uptake within a cancer cohort exists [7, 8, 9], these studies have been limited to small convenience samples from single‐site institutions, hindering generalizability and statistical control due to low power. This limits the ability to detect disparities in vaccine uptake by race/ethnicity or socioeconomic status.

To address these limitations and gain a comprehensive understanding of vaccine hesitancy in the context of cancer, further research is necessary. This entails incorporating larger and more diverse samples to explore specific disparities that may exist in this population. Therefore, we aimed to characterize patterns of early uptake of the COVID‐19 vaccine among cancer survivors and to determine the sociodemographic factors and barriers to vaccine uptake that are associated with survivors who were not fully vaccinated by summer 2021.

## 2 Methods

*Data collection and sample*: Cross‐sectional data for this study were obtained from the “All of Us” research program collected by self‐reported online surveys between May 2018 and July 2022. Briefly, this program is open to US adults aged 18 and over. A signed consent following the Declaration of Helsinki for data collection was obtained from all participants. Data used are de‐identified and available to approved researchers. The All of Us program was approved by the National Institutes of Health (NIH) Institutional Review Board (IRB).

In this study, cancer survivors were defined as those participants who indicated that they had ever been diagnosed with cancer. The *All of Us* program administers multiple surveys to participants. The Basics Survey gathers essential demographic information and must be completed before participants can respond to additional surveys. Two specific surveys were administered by *All of Us*: (1) the Minute Survey, conducted multiple times between June 2021 and March 2022, to assess COVID‐19 vaccine attitudes, beliefs, and uptake, and (2) the COPE Survey, which evaluated the impact of the COVID‐19 pandemic on participants [17]. All cancer survivors with complete questionnaire data about vaccine hesitancy and uptake from June to August 2021 (hereafter referred to as “by summer 2021”) were included in the study cohort.

Over 28,000 cancer survivors in the All of Us program were enrolled in time to be eligible to complete the COPE and Minute surveys. Of these, approximately 27,000 were excluded for having incomplete information on their summer 2021 vaccine uptake, resulting in an ultimate response rate of 1073/28276, or approximately 3.8%. 514 individuals answered questions about their vaccine uptake, vaccine likelihood, and barriers to vaccination, as is used in our results.

### 2.1 Measures

*Demographics*: Individual‐level characteristics included age (at survey completion), sex (male vs. female), race/ethnicity (White, non‐white), state of residence (at survey completion), nativity (born in United States vs. born elsewhere), annual income (using quintiles, the lowest quintile were those who reported income of < 35K vs. ≥ 35K (quintile 2–5)), education (college or more vs. ≤ high school or equivalent), health insurance status (yes vs. no), housing status (own vs. rent/other arrangements), employed (yes vs. no), and whether or not a person is currently undergoing cancer treatment (as determined by both seeing a doctor and receiving treatment).

*Socioeconomic barriers*: Five socioeconomic status (SES) factors (education, income, health insurance status, housing, and employment status) were dichotomized to create a composite measure, in line with previous literature [18]. Cancer survivors with income “≥ 35K,” an education level of “college or more,” health insurance, home ownership, and employment had no SES barriers (score of 0). Additive scores from 1 to 5 were given for facing barriers in any number of the aforementioned categories [18]. To preserve modeling power, scores were truncated at three or more barriers.

*Vaccine likelihood* was assessed using the COVID‐19 Patient Experience (COPE) survey, which was administered from May 2020 to January 2021 prior to widespread accessibility of the COVID‐19 vaccine. Participants were asked about their likelihood of vaccination (“When a COVID‐19 vaccine is available, how likely are you to want to receive vaccination?”) with response options on a 5‐point Likert scale (“Very likely” to “Very Unlikely”).

*Barriers to vaccine uptake*: Factors that made an individual less likely to receive a COVID‐19 vaccine were assessed using the Minute survey on COVID‐19 (MINUTE), which was administered from June 2021 to August 2021, after COVID‐19 vaccines were widely available. Participants were asked what factors might make them less likely to receive the COVID‐19 vaccine and were able to select as many responses as applied to them. These individual responses were grouped into four central categories or themes as barriers to vaccine uptake: 1. *Access Issues* (“I do not want to pay for it,” “The vaccination location is inconvenient,” “There is difficulty making an appointment”), 2. *Medical Distrust* (“It is just a virus/not fatal/not necessary,” “I do not trust the vaccine,” “I am going to let others get it first”), 3. *Perceived Lack of Need* (“I will not get/am never sick,” “I never get vaccinated,” “I am not in a risk group with underlying conditions,” “I have already had COVID‐19,” “My region is not a high risk area”), and 4. *Lack of Information* (“It depends on the risks/adverse events,” “I have not thought about it yet,” “I need more information first”). Additive scores based on the number of responses were created for each of the four categories. To preserve modeling power, scores were truncated (see Table 2), and access issues were transformed to a binary indicator of any access issues vs. no access issues.

*COVID‐19 vaccine uptake by summer 2021*: Participants' vaccination status was self‐reported as part of the Minute Survey on COVID‐19 Vaccines, which was sent to eligible participants who completed the primary consent and the Basics survey. The survey aimed to capture vaccine dosing information on a continuous, rolling basis beginning with vaccine dosing data captured in February 2021 COPE survey. For example, if a patient responded in the February 2021 COPE survey that they received a single dose, then in the subsequent summer 2021 Minute Survey, they were asked about dose two and beyond. Vaccine uptake was categorized as unvaccinated if the participant indicated they had received no doses, initiated if they had one dose, and completed if the participant had at least two doses by the time of survey completion in summer of 2021.

#### 2.1.1 Statistical Methods

A multivariate ordinal logistic regression model was used to test for associations between SES barriers and summer 2021 vaccine uptake, adjusted for age, sex, race/ethnicity, treatment status, access issues, medical distrust, perceived lack of need, and lack of information scores. Per the All of Us data use agreement policy, groups with < 20 participants are shown in descriptive tables as ≤ 20 (%) with a corresponding > 20 (%) category to ensure that another count cannot be used for deriving the exact counts < 20 in the suppressed group. Analyses were performed using R Jupyter Notebooks in the “All of Us” workbench with statistical significance level set at alpha > 0.05. Odds ratios (ORs) with 95% confidence intervals (CI) were reported.

## 3 Results

The study population included 514 cancer survivors from the All of Us Research Program. In total, 73.7% self‐reported as being fully vaccinated with at least two doses of a COVID‐19 vaccine by summer 2021. The mean age of the study population (*n* = 514) was approximately 61 years old. The majority were female (74.3%), self‐identified as non‐Hispanic White (86.8%), and had no SES barriers (54.9%). Most were not currently seeing a doctor and undergoing cancer treatment at the time of survey (73.9%) and resided in states that had expanded their Medicaid programs by 2020 (70.4%). Those unvaccinated by summer 2021 were more likely to face SES barriers (69.0%) compared to those with at least two doses (38.5%) (Table 1).

**TABLE 1:** Demographics by vaccine uptake among All of Us Cancer Survivor Cohort (n = 514).

Demographic	No doses (*N* = 87)	One dose (*N* = 48)	At least two doses (*N* = 379)	Total (*N* = 514)
Age (years)				
Mean (SD)	58.1 (13.6)	60.4 (11.8)	62.3 (11.3)	61.4 (11.8)
Sex assigned at birth, count (%)				
Male	≤ 20 (< 25)	≤ 20 (< 25)	104 (27.4)	132 (25.7)
Female	> 60 (> 75)	> 30 (> 75)	275 (72.6)	382 (74.3)
Race/ethnicity, count (%)				
Non‐white	≤ 20 (< 20)	≤ 20 (< 15)	45 (11.9)	68 (13.2)
White	> 60 (> 80)	> 40 (> 85)	334 (88.1)	446 (86.8)
SES barriers score, count (%)				
0	27 (31.0)	22 (45.8)	233 (61.5)	282 (54.9)
1	< 30 (< 40)	≤ 20 (< 35)	> 25 (> 20)	129 (25.1)
2	≤ 20 (< 25)	≤ 20 (< 20)	49 (12.9)	74 (14.4)
3+	≤ 20 (< 15)	≤ 20 (< 10)	≤ 20 (< 10)	29 (5.6)
State of residence medicaid expansion in 2020, count (%)				
No	> 30 (> 35)	≤ 20 (< 25)	106 (28.0)	152 (29.6)
Yes	> 50 (> 60)	> 30 (> 70)	273 (72.0)	362 (70.4)
Seeing a doctor and receiving treatment for cancer, count (%)				
No	< 60 (< 70)	< 30 (< 70)	280 (73.9)	380 (73.9)
Yes	> 20 (> 20)	≤ 20 (< 25)	99 (26.1)	134 (26.1)

*Note:* Per “All of Us” data use agreement policy, groups with < 20 participants are represented as < 20 (%) with a corresponding > 20 (%) to prevent deriving counts < 20 from other values. Not all percentages sum up to 100%. SES barriers composite score includes: income < 35K, an educational level of “≤ high school or equivalent,” not being insured, not being employed, or not owning a home.

The majority of cancer survivors in the study cohort reported no access issues as barriers to vaccination uptake (Table 2). Survivors with “no doses” by summer 2021 exhibited the highest proportion of medical distrust, with nearly 47.1% reporting at least one medical distrust‐related barrier. Those with “no doses” were also more likely to report perceived lack of need for the vaccine. Out of all possible individual barriers to vaccination regardless of category, the mean number of barriers was greater for cancer survivors in the “no dose” and “one dose” categories, with means of 2.57 ± 1.53 barriers and 2.42 ± 1.53 barriers respectively, compared to a mean number of overall barriers of 2.03 ± 1.42 in the “at least two vaccine doses” group (Table 2).

**TABLE 2:** Barriers to vaccine uptake among All of Us Cancer Cohort.

	No doses (*N* = 87)	One dose (*N* = 48)	At least two doses (*N* = 379)	Total (*N* = 514)
Vaccine likelihood, count (%)				
Very unlikely	> 35 (> 45)	≤ 20 (< 15)	≤ 20 (< 5)	66 (12.8)
Unlikely	≤ 20 (< 20)	≤ 20 (< 15)	≤ 20 (< 5)	33 (6.4)
Unsure	> 25 (> 30)	≤ 20 (< 40)	83 (21.9)	128 (24.9)
Likely	≤ 20 (< 5)	≤ 20 (< 5)	85 (22.4)	89 (17.3)
Very likely	≤ 20 (< 5)	≤ 20 (< 35)	183 (48.3)	198 (38.5)
Barriers to vaccine uptake				
Access issues				
No	> 70 (> 80)	> 35 (> 80)	339 (89.4)	459 (89.3)
Yes	≤ 20 (< 15)	≤ 20 (< 15)	40 (10.6)	55 (10.7)
Medical distrust				
0	> 20 (> 20)	≤ 20 (< 30)	208 (54.9)	248 (48.2)
1	41 (47.1)	20 (41.7)	142 (37.5)	203 (39.5)
2–3	> 20 (> 20)	≤ 20 (< 20)	29 (7.7)	63 (12.3)
Perceived lack of need				
0	> 55 (> 65)	> 35 (> 70)	> 300 (> 80)	421 (81.9)
1	≤ 20 (< 15)	≤ 20 (< 15)	48 (12.7)	68 (13.2)
2–4	≤ 20 (< 20)	≤ 20 (< 5)	≤ 20 (< 5)	25 (4.9)
Lack of information				
0	39 (44.8)	≤ 20 (< 35)	> 75 (> 20)	135 (26.3)
1	20 (23.0)	≤ 20 (< 35)	> 130 (> 30)	175 (34.0)
2–3	28 (32.2)	≤ 20 (< 45)	> 145 (> 35)	204 (39.6)
Overall number of individual barriers (0–7)				
Mean [SD]	2.57 [1.72]	2.42 [1.53]	2.03 [1.30]	2.16 [1.42]

*Note:* Per “All of Us” data use agreement policy, groups with < 20 participants are represented as < 20 (%) with a corresponding > 20 (%) to prevent deriving counts < 20 from other values. Not all percentages sum up to 100%.

Results from the multivariate ordinal logistic regression model showed that race/ethnicity (self‐identified non‐Hispanic White vs. other) was not statistically significantly associated with vaccine uptake (OR (95% CI) = 0.94 (0.51, 1.70)). In the unadjusted model, race was significantly associated with vaccine uptake, with White participants being more likely to complete vaccination than non‐White participants (OR (95% CI = 2.19 (1.31, 3.54)). Residence in a state with Medicaid expansion in 2020 was not statistically significantly associated with vaccine uptake (OR (95% CI) = 1.29 (0.82, 2.01)). Higher SES barriers were found to be significantly associated with lower odds of receiving more vaccine doses, where each additional SES barrier was associated with a 40% reduction in the odds of vaccine uptake (OR (95% CI) = 0.60 (0.48, 0.75)). Higher medical distrust scores were also statistically significantly associated with a 56% decrease in odds of receiving more doses OR (95% CI) = 0.44 (0.32, 0.59), while additional perceived lack of need barriers reduced odds by 39% OR (95% CI) = 0.61 (0.43, 0.87)). Finally, lack of information barrier scores were associated with increased vaccine uptake, where every additional lack of information barrier was associated with a 45% increase in the odds of receiving more doses (OR (95% CI) = 1.45 (1.13, 1.86)) (Table 3).

**TABLE 3:** Multivariable ordinal logistic regression of vaccine uptake by personal characteristics and barriers among cancer survivors.

	Unadjusted			Adjusted		
	OR	95% confidence interval		OR	95% confidence interval	
Age (years)	1.03	1.02	1.05	1.02	1.00	1.03
Female (vs. male)	0.51*	0.34*	0.74*	0.75	0.44	1.25
Race (white vs. non‐white)	2.19*	1.31*	3.54*	0.94	0.51	1.70
Current treatment (yes vs. no)	1.06	0.73	1.56	1.15	0.71	1.88
Medicaid expansion (yes vs. no)	1.28	0.90	1.81	1.29	0.82	2.01
SES barriers score	0.56*	0.47*	0.67*	0.60*	0.48*	0.75*
Barriers to vaccine likelihood						
Access issues (yes vs. no)	0.90	0.53	1.84	1.62	0.83	3.34
Medical distrust score	0.43*	0.33*	0.56*	0.44*	0.32*	0.59*
Perceived lack of need score	0.49*	0.36*	0.66*	0.61*	0.43*	0.87*
Lack of information score	1.40*	1.10*	1.77*	1.45*	1.13*	1.86*

*Note:* Significant *p* values *< 0.05. SES barriers composite score includes: income < 35K, an educational level of “≤ high school or equivalent,” not being insured, not being employed, or not owning a home. *Adjusted model*: Adjusted odds ratios (OR) for: SES barriers, medical distrust, perceived lack of need, lack of information, current cancer treatment status, gender, and residence in a state with Medicaid expansion. *Unadjusted model*: Raw association unadjusted for any variables.

## 4 Discussion

Using data from the All of Us Research Program, this study found that a high proportion (73.7%) of cancer survivors in our study self‐reported having at least two doses of a COVID‐19 vaccine by summer 2021. In contrast, approximately 54% of the general US population was fully vaccinated by the end of August 2021, highlighting the relatively high vaccination rate among cancer survivors in our sample [19]. This suggests that efforts to vaccinate cancer survivors have yielded positive results; however, future efforts should focus on preventing widening disparities. Our findings revealed that sociodemographic factors such as race/ethnicity, gender, and residence in a state with Medicaid expansion were not statistically significantly associated with receiving more COVID‐19 vaccine doses. However, SES barriers were found to have a substantial impact on odds of vaccination. Higher medical distrust and perceived lack of need barrier scores were also statistically significantly associated with lower odds of vaccination as prevalent barriers to vaccination, while lack of information barrier scores were associated with greater odds of vaccination. These findings emphasize the importance of addressing socioeconomic disparities in conjunction with building trust in high‐risk groups to enhance vaccination rates. Public health campaigns should focus on educating cancer survivors about the ongoing need for vaccination, even after the initial phases of the pandemic, to prevent severe illness and death. Tailoring messaging to emphasize the specific benefits of vaccination for immunocompromised individuals, including those with cancer, may help to counteract the perceived lack of need.

### 4.1 Socioeconomic Status Barriers and Vaccine Uptake

Our multivariate ordinal logistic regression model suggested that cancer survivors who experience more SES barriers had a lower likelihood of receiving more COVID‐19 vaccine doses by summer 2021. These findings align with previous research indicating that socioeconomic status can play a role in vaccination behaviors. For example, a study among cancer survivors from neighborhoods in Ontario, Canada found that those with the lowest socioeconomic status and highest material deprivation had a 20% lower vaccination rate compared to those in more advantaged neighborhoods [20]. The influence of socioeconomic disparities on vaccination is even further highlighted by findings on the speed of vaccine uptake, where patients in the lowest socioeconomic strata required more time to attain full vaccination and booster doses compared to their more advantaged counterparts [20]. Similarly, in another study of cancer survivors who had tested positive for COVID‐19 in 2020, lower educational attainment and higher unemployment rates were associated with lower COVID‐19 vaccination rates [21].

It is noteworthy that our study found no significant racial/ethnic disparities in early vaccine uptake among All of Us cancer survivors in the adjusted model despite the presence of disparities in the unadjusted model. After adjusting for SES barriers, this racial disparity was no longer significant, suggesting that existing racial/ethnic disparities in vaccine uptake may instead be explained by the presence of SES barriers. This contrasts with previous studies showing racial/ethnic disparities in COVID‐19 vaccine intention and initiation. For example, a nationwide survey in December 2020 assessing the likelihood of COVID‐19 vaccination among past or current patients with cancer found that those unlikely to accept the vaccine were more often Black, Indigenous, or People of Color [22]. Similarly, among Medicare beneficiaries, Hispanic and Black respondents were also less likely to have received the COVID‐19 vaccine compared to non‐Hispanic White respondents [23]. The absence of racial/ethnic disparities in our study, however, may also be explained by the composition of the self‐selected study cohort, wherein less educated and lower income cancer survivors who are more likely to have limited access to health care are less represented. One study indicated a minority of cancer survivors in the All of Us Research Program encountered difficulty paying for healthcare in the past 12 months [24], whereas these financial challenges are likely more prevalent among cancer survivors in the broader population.

In a study assessing vaccination trends by race/ethnicity and socioeconomic status, household income, education level, and health insurance type mediated the racial differences in several vaccinations (influenza, pneumococcal, tetanus, diphtheria, acellular pertussis, and zoster) among U.S. adults ≥ 65 years of age [25]. Additionally, cancer survivors with low income may prioritize daily living needs, such as food, housing, household bills, over healthcare services and are also more likely to receive COVID‐19 vaccination when provided paid sick leave [26, 27]. Thus, when faced with the choice between taking time off from work without pay or not getting vaccinated, individuals with lower income and greater financial constraints may be more likely to delay or forgo receiving the COVID‐19 vaccine altogether.

### 4.2 Barriers to Vaccine Uptake Among the Cancer Survivor Community

#### 4.2.1 Medical Distrust

Our analysis revealed that cancer survivors with no vaccine doses reported the highest proportion of medical distrust‐related barriers compared to those with one or more doses, highlighting medical distrust as significant hurdle to vaccination. In one study among hematologic malignancy patients, there was a positive correlation between vaccination in June 2021 and trust levels in oncologists, federal agencies, and pharmaceutical companies, which can all serve as primary distributors of the vaccine while underscoring its importance [28]. Cancer survivors also hold a different set of trust‐related concerns compared to the general population, in part because they were excluded from COVID‐19 vaccine trials, resulting in a lack of data on vaccine safety and efficacy for this group [29]. Those with concerns of the vaccine interfering with their treatment are also significantly more likely to refuse the vaccine [30].

#### 4.2.2 Lack of Information Barriers

Results from the multivariate ordinal logistic regression model revealed a positive association between the number of lack of information barriers and the odds of receiving more COVID‐19 vaccine doses by summer 2021. Contrary to the initial expectation that perceived insufficient information on the COVID‐19 vaccine would deter vaccine uptake, individuals expressing such sentiments might take proactive measures to investigate their reservations and likely gain confidence in vaccination, ultimately leading vaccination. Many individuals also do not immediately adopt vaccines upon initial recommendations and require time to process information and address their concerns before deciding to vaccinate. Perceptions of COVID‐19 vaccines and readiness to vaccinate evolved over time, particularly during the early vaccine rollout in January 2021, when vaccine hesitancy was at its peak. According to a report by the Kaiser Family Foundation, in February 2021, 22% of US adults expressed a desire to “wait and see” before getting vaccinated [31]. By May 2021, this hesitancy shifted, with 12% still wanting to “wait and see.” At that point, 68% of US adults had received at least one dose of the vaccine series [32]. Furthermore, the endorsement of vaccination by healthcare professionals over time may have played a crucial role in the decision‐making process, underscoring the influential role of healthcare providers in information dissemination within this population [33].

### 4.3 Strengths and Limitations

An important strength of this study is the comprehensive nature of early vaccine uptake data that go beyond a binary analysis of individuals who were vaccinated versus unvaccinated and distinguishes between individuals with no doses, a single dose, and those with at least two doses by summer 2021. Furthermore, utilizing data from the All of Us Research Program, known for its diverse representation of underrepresented minority populations and sociodemographic characteristics, enhances our findings' generalizability to cancer survivors with similar SES and barriers to vaccine uptake in the United States.

Our study was limited, however, by the number and composition of cancer survivors with questionnaire data. One limitation is the limited diversity in a self‐selected sample cohort that is largely female, non‐Hispanic white, and of low SES barriers may not fully represent the broader US population. While over 28,000 cancer survivors enrolled in the All of Us Research Program were eligible to answer the COPE and Minute Surveys, about 1000 answered questions about their summer 2021 vaccine uptake, and only 514 answered questions about vaccine uptake, vaccine likelihood, and barriers to vaccination. To sufficiently power our models, we reduced the number of categories for many variables, including vaccination barriers and race/ethnicity.

### 4.4 Implications and Future Directions

This study offers valuable implications for the promotion of early COVID‐19 vaccine uptake among cancer survivors. With the COVID‐19 vaccines being reimagined as an annual immunization effort targeting specific variants of the virus [34], it is crucial to address the specific barriers faced by this population to promote immunity and decrease hospitalizations and deaths on a continuous basis. The findings emphasize the need for tailored interventions that target socioeconomic challenges such as economic disparities, educational barriers, job opportunities, affordable housing, and accessible health insurance for cancer survivors. Addressing socioeconomic barriers alone, however, is insufficient; there is a need to prioritize trust‐building initiatives, such as targeted education and endorsement from oncology providers, primary health care providers, nurse practitioners, physician assistants, social workers, and public health officials to promote equitable COVID‐19 vaccination.

Future studies should examine a larger and more diverse cohort of cancer survivors to validate these findings as the All of Us Research Program enrolls more participants. Additionally, future studies should analyze socioeconomic barriers individually, namely low income, education level, and health insurance status, in relation to ongoing COVID‐19 booster vaccination. Investigating the individual contributions of these factors will provide a more nuanced understanding of which factor holds more salience in influencing vaccine adoption within this population.

## 5 Conclusion

In conclusion, our study highlights the multiple factors that impact early vaccine uptake among cancer survivors. It underscores the importance of concurrently addressing socioeconomic disparities, while building and fostering trust through appropriate information dissemination that stresses the advantages of vaccination for cancer survivors and other high‐risk groups. Future research may benefit from assessing individual socioeconomic barriers, such as income, education, and employment, to pinpoint the factors exerting more influence on early COVID‐19 vaccine uptake. These insights can inform the development of targeted public health initiatives to aid in enhancing efficiency of vaccination campaigns for vulnerable groups as new boosters become available.

## Author Contributions

**Nikta Saeedi:** conceptualization (equal), formal analysis (equal), methodology (equal), writing – original draft (lead), writing – review and editing (lead). **Angel Arizpe:** conceptualization (equal), methodology (equal), writing – original draft (equal), writing – review and editing (equal). **Katelyn J. Queen:** conceptualization (equal), formal analysis (equal), methodology (equal), writing – original draft (equal), writing – review and editing (equal). **Jennifer Tsui:** conceptualization (equal), data curation (equal), funding acquisition (equal), writing – review and editing (equal). **Sue Kim:** conceptualization (equal), data curation (equal), funding acquisition (equal), writing – review and editing (equal). **Brian Z. Huang:** conceptualization (equal), data curation (equal), funding acquisition (equal), writing – review and editing (equal). **Albert J. Farias:** conceptualization (lead), data curation (equal), formal analysis (equal), funding acquisition (lead), methodology (equal), writing – original draft (equal), writing – review and editing (equal).

## Conflicts of Interest

The authors declare no conflicts of interest.

## Précis

Socioeconomic barriers are significantly associated with disparities in COVID‐19 vaccine uptake among cancer survivors. Addressing these barriers is crucial for enhancing future vaccination rates.

## Data Availability

The data for this study are derived from a public domain. Data that support the findings are available from the All of Us Research Program and can be accessed through the Researcher Workbench (https://workbench.researchallofus.org/login) as per the informed consent of program participants.
